# Advanced Therapeutic Approaches for Metastatic Ovarian Cancer

**DOI:** 10.3390/cancers17050788

**Published:** 2025-02-25

**Authors:** Soohyun Choe, Minyeong Jeon, Hyunho Yoon

**Affiliations:** 1 Department of Medical and Biological Sciences, The Catholic University of Korea, Bucheon 14662, Republic of Korea; cshyun16@catholic.ac.kr (S.C.); someyou25@catholic.ac.kr (M.J.); 2 Department of Biotechnology, The Catholic University of Korea, Bucheon 14662, Republic of Korea

**Keywords:** ovarian cancer, peritoneal metastasis, tropism, signaling pathway, tumor microenvironment, immunotherapy

## Abstract

Ovarian cancer is the fifth leading cause of cancer-related death among women, which is one of the most common gynecological cancers worldwide. Although several cytoreductive surgeries and chemotherapies have been attempted to address ovarian cancer, the disease still shows poor prognosis and survival rates due to prevalent metastasis. Peritoneal metastasis is recognized as the primary route of metastatic progression in ovarian cancer. It causes severe symptoms in patients, but it is generally difficult to detect at an early stage. Current anti-cancer therapy is insufficient to completely treat metastatic ovarian cancer due to its high rates of recurrence and resistance. Therefore, developing strategies for treating metastatic ovarian cancer requires a deeper understanding of the tumor microenvironment (TME) and the identification of effective therapeutic targets through precision oncology. Given that various signaling pathways, including TGF-β, NF-κB, and PI3K/AKT/mTOR pathways, influence cancer progression, their activity and significance can vary depending on the cancer type. In ovarian cancer, these pathways are particularly important, as they not only drive tumor progression but also impact the TME, which contributes to the metastatic potential. The TME plays a critical role in driving metastatic features in ovarian cancer through altered immunologic interactions. Recent therapeutic advances have focused on targeting these distinct features to improve treatment outcomes. Deciphering the complex interaction between signaling pathways and immune populations contributing to metastatic ovarian cancer provides an opportunity to enhance anti-cancer efficacy. Hereby, this review highlights the mechanisms of signaling pathways in metastatic ovarian cancer and immunological interactions to understand improved immunotherapy against ovarian cancer.

## Simple Summary

Ovarian cancer is one of the deadliest cancers in women because it often spreads to the peritoneum, leading to severe symptoms and making early detection difficult. Even with advancements in surgery and chemotherapy, the cancer frequently comes back, and treatment becomes less effective, especially after it has spread. This has created an urgent need for better treatment options. The tumor’s surrounding environment, known as the tumor microenvironment, plays a key role in the growth and spread of ovarian cancer. It includes various immune cells and important signaling pathways, such as TGF-β, NF-κB, and PI3K/AKT/mTOR, which help the cancer survive and grow. Understanding how these pathways work together can help in the development of more effective and targeted treatments. This review includes the complex mechanisms behind metastatic ovarian cancer and highlights combination therapy as a promising approach to improving treatment outcomes.

## 1. Introduction

Ovarian cancer (OC) is one of the most common gynecologic cancers and has the highest mortality rate due to prevalent malignant metastasis [1]. A hallmark of OC metastasis is its preferential spread to the peritoneal cavity, a process referred to as peritoneal metastasis [2]. Unlike hematogenous dissemination common in other cancers, OC cells dissociate from the primary tumor, persist within the ascitic fluid, and adhere to the mesothelial layer of the peritoneum [3]. This process is facilitated by versatile interactions between tumor cells and the peritoneal microenvironment, including mesothelial cells, immune infiltrates, and extracellular matrix (ECM) components [4,5]. These metastatic mechanisms make peritoneal dissemination an important contributor to the poor prognosis of OC [5]. Despite continuous developments in diagnostic techniques, surgical interventions, and the advancement of therapeutic approaches, the five-year survival rate for OC remains unsatisfactory [6]. Currently, standard treatment, which combines maximal cytoreductive surgery and postoperative platinum-based chemotherapy, frequently leads to high recurrence rates and limited long-term benefits [7,8]. Moreover, the cumulative toxicity of chemotherapy often results in severe adverse effects, further deteriorating patient outcomes [9]. Considering these challenges, it is crucial to develop advanced therapeutic strategies targeting metastatic OC [10].

Metastatic OC arises from intricate signaling networks that not only regulate tumor progression but also confer resistance to existing treatments [11,12]. The transforming growth factor-β (TGF-β) pathway exerts contrasting roles in OC pathogenesis, serving as a tumor suppressor in the initial stages of the disease while promoting processes such as epithelial-to-mesenchymal transition (EMT) and immune evasion during advanced metastatic progression [13]. The loss of tumor-suppressive TGF-β signaling promotes EMT, a critical process in peritoneal dissemination and metastasis [14]. In chemoresistant OC cells, TGF-β signaling is often impaired, particularly through the disruption of the suppressor of mothers against decapentaplegic 3 (SMAD3) phosphorylation, reducing its tumor-suppressive effects [15]. Beyond its canonical SMAD-mediated pathway, TGF-β activates non-SMAD pathways, such as phosphoinositide 3-kinase (PI3K)/protein kinase B (AKT)/mammalian target of rapamycin (mTOR) and mitogen-activated protein kinase (MAPK)/extracellular signal-regulated kinase (ERK) cascades, which are essential for sustaining tumor cell survival, proliferation, and invasive traits [16,17,18]. Notably, the hyperactivation of the PI3K/AKT/mTOR axis is a representative feature of OC, resulting in chemoresistance by enhancing cellular growth, metabolic reprogramming, and autophagy [19,20]. This multifaceted participation of signaling transductions highlights its therapeutic significance in managing OC progression. The nuclear factor of the kappa-light chain of the enhancer-activated B cell (NF-κB) pathway also plays a pivotal role in OC metastasis by building the immunosuppressive tumor microenvironment (TME) [21]. Tumor-associated macrophages (TAMs) release tumor necrosis factor-alpha (TNF-α), creating a positive feedback loop that sustains NF-κB activation [22]. It promotes tumor metastasis, immune evasion, and cancer stem cell proliferation [23]. Elevated NF-κB signaling correlates with increased tumor aggressiveness and poor clinical outcomes, indicating the potential of the signaling pathway as a therapeutic target. Concertedly, it is sophisticated that the critical roles of TGF-β, NF-κB, and PI3K/AKT/mTOR signaling transductions involve inducing metastatic progression and resistance in OC, underlining the desire for improved therapeutic strategies targeting these networks.

Although immunotherapy has rapidly evolved as an anti-cancer treatment, its efficacy in OC has been limited, with clinical trials often reporting suboptimal response rates [24]. Conventional immunotherapy such as immune checkpoint inhibitor fails to adequately overcome the immunosuppressive barriers established by the OC TME [25]. It has prompted interest in combination therapies that integrate immunotherapy with targeted inhibitors of major signaling pathways, including TGF-β, NF-κB, and PI3K/AKT/mTOR. Such approaches aim to disrupt the pro-metastatic and immunosuppressive signaling networks while enhancing anti-tumor immune responses [26].

In this review, we discuss the molecular mechanisms associated with OC metastasis, focusing on the vital signaling pathways that produce tumor progression and therapeutic resistance. Furthermore, this paper explores emerging strategies to address these pathways, with an emphasis on combination therapies that are promising for overcoming the present limitations of current treatments.

## 2. Ovarian Cancer Tropism

**Figure 1:**
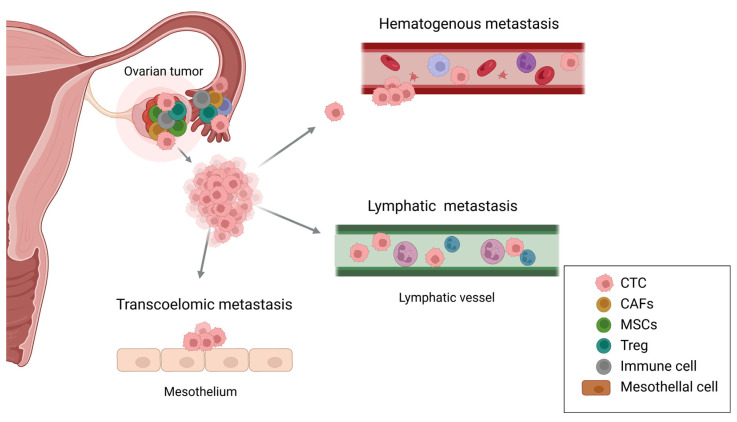
Mechanisms of ovarian cancer tropism. OC metastasizes through multiple routes, including transcoelomic, hematogenous, and lymphatic dissemination. Transcoelomic metastasis involves the direct spread of cancer cells to the peritoneal cavity, where they attach to the mesothelial lining. Hematogenous metastasis occurs when CTCs enter the bloodstream, enabling distant organ dissemination. Lymphatic metastasis implies cancer cells spreading via lymphatic vessels. The TME comprises CAFs, MSCs, Tregs, immune cells, and mesothelial cells, which overall contribute to tumor progression and immune evasion. They play important roles in promoting metastasis through interactions within TME. OC, ovarian cancer; CTC, circulating tumor cell; CAFs, cancer-associated fibroblasts; MSCs, mesenchymal stem cells; Treg, regulatory T cell; TME, tumor microenvironment.

OC is characterized by its unique tropism and metastatic behavior, leading to poor survival and high recurrence rates. Understanding tropism in cancer cells provides valuable insights into the mechanisms of metastasis, particularly in explaining why cancer cells preferentially metastasize to specific organs [27]. Metastatic organotropism is regulated by molecular signaling pathways, tumor-intrinsic factors, and interactions with the TME [28]. OC metastasis often spreads from the primary tumor site to adjacent organs through three major routes: transcoelomic, lymphatic, and hematogenous dissemination [2] (Figure 1). Transcoelomic metastasis, facilitated by peritoneal fluid or ascites, is known to be predominant [29]. Due to the proximity of the peritoneal cavity to the ovaries and the absence of a physical barrier between the tumor and the peritoneum, this provides a preferential route for metastasis to the omentum or peritoneum [2]. Lymph nodes connected to tumor vascularization can act as drainage sites for cancer cells, allowing them to shed, circulate, and spread via the lymphatic system [30]. Hematogenous metastasis is the least common route of OC dissemination and is associated with the presence of circulating tumor cells in the bloodstream. However, the lack of effective methods for detecting circulating tumors has hindered a comprehensive understanding of the mechanisms underlying hematogenous spread [31].

As mentioned above, peritoneal metastasis, the most common metastatic site in OC, accounts for the majority of OC cases and involves specific processes, including separation from the primary tumor, spread to the peritoneum, colonization, and progression into metastatic tumors in the peritoneum [32]. The peritoneal membrane, composed of epithelial-like peritoneal mesothelial cells (PMCs) supported by an ECM-rich stroma, can play an essential role in tumor colonization [33]. This process is driven by integrins and ECM remodeling, which provide essential metabolic substrates, along with the immune landscape of the peritoneal cavity that shapes the metastatic niche [2,34]. The peritoneal immune microenvironment is dominated by cancer-associated fibroblasts (CAFs), TAMs, regulatory T cells (Tregs), and myeloid-derived suppressor cells (MDSCs), all of which contribute to immunosuppression [35,36]. CAFs contribute to OC peritoneal metastasis by promoting immune cell recruitment, ECM remodeling, and the secretion of cytokines and chemokines that rebuild the TME. In malignant ascites, CAFs express certain genes related to the immune system, such as complement factors, chemokines (e.g., CXCL1/2/10/12), and cytokines (e.g., IL6, IL10), which subsequently enhance tumor formation, invasion, and progression through the activation cadherins and integrins [36]. TAMs in ascites facilitate tumor progression via ECM degradation, angiogenesis, and immune evasion through cytokine secretion such as IL-10 and TGF-β [37]. Tregs suppress cytotoxic T cell activity of immune effector cells, thereby enabling tumor survival and proliferation [38]. Therefore, these immune populations generally induce a permissive environment that facilitates peritoneal metastasis. Given that OC exhibits versatile immune dysregulation and metastatic organotropism, it is required to elucidate the pivotal role of various signaling pathways regarding OC tropism and metastasis. Additionally, this understanding can offer potential therapeutic targets for addressing peritoneal metastasis.

## 3. Metastatic Signaling Pathways in Ovarian Cancer

The metastatic progression of OC is regulated by a complex network of signaling pathways related to EMT, immune evasion, and tumor development [39]. EMT, a hallmark of metastasis, enables epithelial cancer cells to acquire mesenchymal traits that promote their mobility and invasiveness [40]. This process is closely correlated with immune regulation and metabolic reprogramming within the TME, further reinforcing the metastatic potential of cancer cells [41]. Given that EMT is essential for cancer cells to migrate and settle in distant sites such as the peritoneum, understanding EMT-associated signaling pathways is critical for unraveling the mechanisms of peritoneal dissemination in OC.

### 3.1. TGF-β Signaling Pathway

The TGF-β family plays a dual role in tumorigenesis, acting as a tumor suppressor in the early stages and as a promoter of metastasis in advanced stages [13]. In OC, TGF-β signaling functions as an early EMT inducer that regulates cancer cell metastasis and development [42]. EMT provides metastatic characteristics through the reversible transition of epithelial cells into mesenchymal cells. This process is characterized by the downregulation of transcriptional epithelial markers, such as E-cadherin, and the upregulation of mesenchymal markers, such as N-cadherin and vimentin [43]. TGF-β can impair the immune responses of NK cells, cytotoxic T lymphocytes (CTLs), and CD8^+^ T cells, allowing cancer cells to evade immune surveillance [44]. Within the TME, activated TGF-β signaling polarizes macrophages and neutrophils into TAMs, further facilitating immune evasion. Beyond immune suppression, TGF-β induces CAFs, which recruit tumor-promoting factors that, in turn, accelerate cancer progression [17]. TGF-β also contributes to tumor invasion and migration by promoting the detachment of OC cells from the primary tumor, especially in the absence of TGF-β receptors and SMAD proteins [17,44]. Stimulated TGF-β/SMAD signaling enhances peritoneal invasion through the secretion of matrix metalloproteinases (MMPs), which degrade the ECM and enable cancer cells to invade surrounding tissues [45]. Additionally, MMPs activate various growth factors in the ECM, contributing to tumor proliferation and angiogenesis [46]. Importantly, TGF-β-induced EMT through both SMAD and non-SMAD pathways is a canonical process in metastasis, as it produces transcription modulators, such as SNAIL, ZEB, and TWIST, and triggers other EMT-related signaling pathways [47]. TGF-β/SMAD signaling also enhances the PI3K/AKT pathway, promoting cancer invasion and migration [48]. Furthermore, the MAPK cascade is activated, contributing to tumorigenesis as dephosphorylation of SMAD at the MAPK site reduces its nuclear stability and activity, counteracting the tumor-suppressive effects of TGF-β [48]. The interaction between OC and its EMT induced by TGF-β not only enhances cellular invasiveness but also suppresses immune responses; thus, understanding the pivotal mechanisms underlying this process is crucial for identifying therapeutic targets aimed at the metastatic downstream pathways in OC.

### 3.2. NF-κB Signaling Pathway

The NF-κB pathways is a central regulator of inflammation, cell survival, and proliferation, playing a critical role in driving cancer metastasis, including OC dissemination and peritoneal metastasis [49]. NF-κB contributes to tumorigenesis in various ways. When damaged DNA strands are detected, NF-κB promotes tumorigenesis by activating mutagenic enzymes, such as cytidine deaminase, which induces mutations by converting cytosine to thymine during cell cycle progression [49]. These mutations provide cancer cells with the genetic plasticity required for adaptation and survival [50]. Furthermore, NF-κB promotes EMT by regulating EMT-associated transcription factors, such as SNAIL and TWIST, thereby enhancing the migratory capacity and stemness of cancer cells [51]. In addition, NF-κB drives the secretion of cytokines such as vascular endothelial growth factor (VEGF) and TGF-β. VEGF induces angiogenesis and vascular remodeling, enabling cancer cells to disseminate into the peritoneum. Concurrently, TGF-β upregulates metastatic genes and MMPs, amplifying ECM remodeling and enhancing hypoxic conditions that support tumor progression [49,52]. This dual function establishes a pro-tumorigenic environment that facilitates OC metastasis. Within the TME, NF-κB signaling is activated by pro-inflammatory cytokines such as TNF-α and IL-1β, which are prevalent in the peritoneal microenvironment. This persistent activation triggers NF-κB-driven inflammatory responses and cytokine production, maintaining a TME conducive to immune evasion, angiogenesis, and ECM remodeling, collectively driving ovarian cancer metastasis [53]. Additionally, NF-κB signaling in the TME fosters immune evasion by polarizing macrophages into TAMs and inducing CAFs, both of which facilitate peritoneal dissemination [54,55]. Thus, NF-κB signaling interacts with EMT-associated transcription factors, further serving the metastatic potential of OC.

### 3.3. PI3K/AKT/mTOR Signaling Pathway

The PI3K/AKT/mTOR pathway is frequently dysregulated in OC, playing a significant role in cell survival, proliferation, resistance to apoptosis, and chemotherapeutic resistance [56]. Malfunction in this pathway supports tumor growth and adaptability, particularly under the metabolic and immune pressures of the OC TME [57]. Key aberrant alterations in OC include PIK3CA amplification and PTEN loss through deletion or epigenetic silencing [57]. Studies have demonstrated that components of this pathway, such as phosphorylated AKT and mTOR-associated proteins, are highly expressed in aggressive tumors and are associated with poor prognosis [20]. The PI3K/AKT/mTOR signaling also influences immune interactions and promotes tumor progression within the TME [58]. In high-grade OC, CAFs release hepatocyte growth factor (HGF), activating the PI3K/AKT axis to enhance OC proliferation, including integrin-mediated interactions [59]. It motivates tumor cell adhesion to the mesothelial lining of the peritoneum, a crucial step in initiating peritoneal metastasis [60]. Subsequently, genetic alterations such as mutations or upregulated expression and activity in PIK3CA, AKT1, and mTOR are significantly observed in OC, underscoring their role in promoting oncogenic signaling [20,61]. Moreover, it facilitates metabolic reprogramming, enabling cancer cells to effectively utilize lipids from omental adipocytes as a primary energy source, a pivotal process for their survival under nutrient-deprived conditions [62,63]. Furthermore, the PI3K/AKT/mTOR pathway regulates angiogenesis by enhancing VEGF expression, which ensures nutrient supply while supporting the formation of blood vessels and oxygen delivery to establish secondary metastatic sites [64]. The activation of AKT directly stabilizes SNAIL, a master regulator of EMT, and promotes the suppression of E-cadherin, thereby reinforcing mesenchymal traits [40]. The role of PI3K/AKT/mTOR in modulating immunoreaction, including the suppression of cytotoxic T cell responses and the recruitment of immunosuppressive TAMs, further contributes to OC progression and metastasis [58]. Therefore, the PI3K/AKT/mTOR pathway is involved in EMT-related pathways in cancer cells, controlling cytoskeletal reorganization, enhanced cellular motility, and tumor aggressiveness which are hallmarks of metastatic competence [20,65].

**Figure 2:**
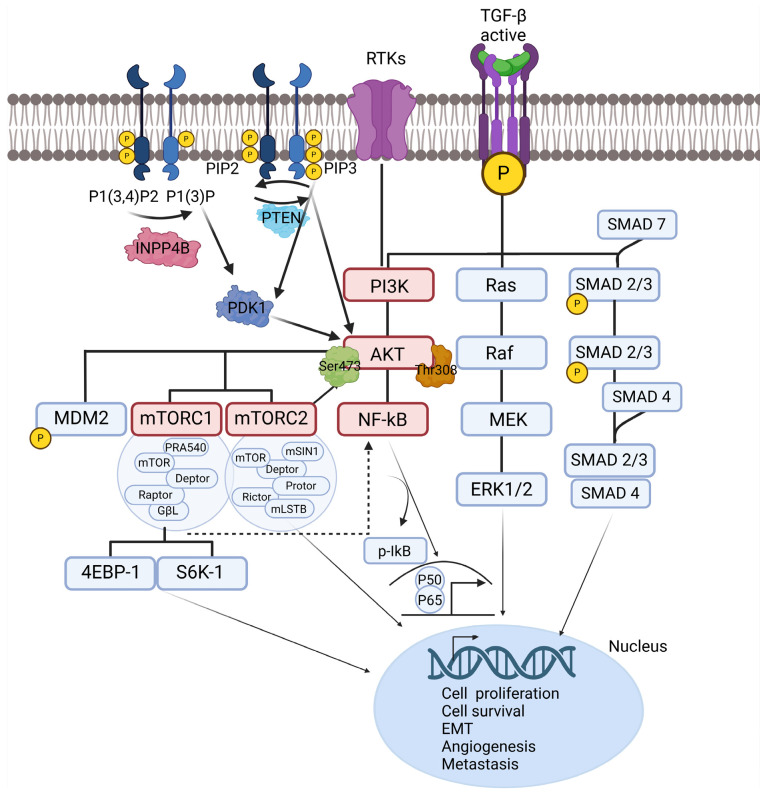
Key signaling pathways involved in ovarian cancer metastasis. It illustrates the molecular interactions among major signaling pathways driving OC metastasis, including TGF-β, NF-κB, and PI3K/AKT/mTOR pathways. Upon activation, TGF-β receptors phosphorylate SMAD proteins, which then associate with SMAD4 and translocate to the nucleus to regulate gene expression, enhancing EMT, angiogenesis, and metastasis. SMAD7 acts as a negative regulator, inhibiting this signal. RTKs and TGF-β activate PI3K, leading to the generation of PIP3 and the recruitment of AKT. Stimulated AKT phosphorylates downstream targets, including mTOR complexes, which modulate cell growth, proliferation, and survival via effectors like 4EBP-1 and S6K-1. PTEN negatively regulates this pathway by dephosphorylating PIP3. Crosstalk between the PI3K/AKT and NF-κB pathways amplifies metastatic processes. AKT activation facilitates NF-κB signaling by promoting the phosphorylation and degradation of IκB, releasing the p50/p65 complex for nuclear translocation. This cascade controls the expression of genes involved in inflammation, immune evasion, EMT, and stemness. OC, ovarian cancer; TGF-β, transforming growth factor-β; NF-κB, nuclear factor of kappa-light chain of enhancer-activated B cells; PI3K, phosphoinositide 3-kinase; AKT, protein kinase B; mTOR, mammalian target of rapamycin; RTK, receptor tyrosine kinase; PIP2, phosphatidylinositol 4,5-bisphosphate; PIP3, phosphatidylinositol (3,4,5)-triphosphate; PTEN, phosphatase and tensin homolog; INPP4B, inositol polyphosphate 4-phosphatase B; PDK1, phosphoinositide-dependent protein kinase 1; SMAD, suppressor of mothers against decapentaplegic; MEK, mitogen-activated protein kinase; ERK, extracellular signal-regulated kinase.

Understanding the metastatic signaling pathways in OC provides a detailed perspective into the molecular mechanisms regarding peritoneal metastasis. Overall, TGF-β, NF-κB, and PI3K/AKT/mTOR pathways closely cooperate within the TME as a complex network, influencing EMT, cell proliferation, and immune evasion (Figure 2). Therefore, targeting these pathways has the potential to disrupt the metastatic cascade and improve clinical outcomes for OC patients.

## 4. Therapeutic Applications for Ovarian Cancer Metastasis

Despite advancements in immunotherapy, the clinical management of metastatic OC remains challenging mainly due to the immunosuppressive TME of OC [66]. Currently, only the use of an antibody targeting cytotoxic T lymphocyte-associated protein 4 (CTLA-4) or programmed cell death (PD-1)/programmed cell death ligand (PD-L1) has shown limited efficacy in OC along with poor outcomes [67]. Thus, there is an urgent need to develop advanced treatment strategies, such as combining traditional immunotherapies—like immune checkpoint inhibitors—with other anti-cancer treatments, including targeted therapies (e.g., TGF-β or PI3K/AKT/mTOR inhibitors) and chemotherapy. In order to address versatile therapeutic implications, a comprehensive understanding of widely used strategies is essential.

### 4.1. Current Therapeutic Approaches in Ovarian Cancer Metastasis

OC remains a significant clinical challenge due to its aggressive nature and resistance to treatment, especially when it progresses to metastasis. Mitotic-poisoning agents, platinum-based compounds, and poly (ADP-ribose) polymerase (PARP) inhibitors are widely used therapeutic strategies for metastatic OC, each addressing different yet interrelated pathways that drive tumor progression and metastasis [68,69]. Mitotic-poisoning agents, such as paclitaxel and docetaxel, function as microtubule-stabilizing drugs to promote microtubule polymerization and disrupt microtubule dynamics, thereby inhibiting proper mitotic spindle formation and inducing cell cycle arrest in the metaphase stage [70]. This disruption prevents cancer cells from completing mitosis, ultimately leading to apoptosis [71]. In metastatic OC, paclitaxel is commonly used in combination with platinum-based compounds to enhance therapeutic efficacy [72]. Despite its effectiveness, the prolonged use of mitotic inhibitors can lead to drug resistance through mechanisms such as increased expression of efflux pumps and alterations in microtubule-associated proteins [73].

Platinum-based compounds, including cisplatin and carboplatin, are pivotal in chemotherapy regimens for both primary and metastatic OC, interfering with DNA replication and enhancing cancer cell death [72]. These agents induce DNA crosslinking, resulting in replication stress and the activation of apoptotic pathways [74]. However, the emergence of platinum resistance, often due to enhanced DNA repair mechanisms, remains a significant hurdle in long-term treatment outcomes. To counteract this resistance, platinum-based chemotherapy is often combined with other targeted therapies, including PARP inhibitors [75].

PARP inhibitors represent a breakthrough in the cancer-targeting treatment of metastatic OC, particularly in patients with BRCA1/2 mutations or homologous recombination deficiency (HRD) [76]. By inhibiting the PARP enzyme, these drugs prevent the repair of single-strand DNA break, leading to certain lethality in HRD-positive cancer cells [77]. Olaparib, rucaparib, and niraparib have demonstrated substantial efficacy in prolonging progression-free survival in OC patients, particularly as maintenance therapy following platinum-based treatment [78]. Furthermore, combination strategies integrating PARP inhibitors with immune checkpoint inhibitors are being actively explored to enhance anti-tumor responses and overcome acquired resistance [79].

### 4.2. Recent Advanced Combination Immunotherapy in Ovarian Cancer Metastasis

Recent studies on NK cell therapy using oncolytic viruses demonstrated a promising anti-cancer immune reaction [80]. While TGF-β in ascites exhibits an immunosuppressive effect in OC, the proper function of NK cells is compromised by the downregulation of NK cell-activating receptors [81]. Therefore, recent research has focused not only on the direct killing of cancer cells but also on boosting immune cell activity. Oncolytic viruses infect cancer cells and stimulate “danger signals”, which help construct an immunogenic TME and regulate inflammatory and immunomodulatory cytokines with transduction molecules [82]. According to studies with experimental cancer models, vesicular stomatitis virus (VSV) and reovirus, both recombinant oncolytic viruses, have successfully induced NK cell activation, resulting in an increased survival rate in peritoneal OC models compared to single-agent therapies [80]. Notably, among the two viruses, the infection of VSV-enhanced antigen presentation capabilities and the activation of IFN-γ-generating NK, CD8^+^, and CD4^+^ T cells accelerated the anti-cancer immune response [80]. As a result, combination therapy of oncolytic virus and NK cell immunotherapy presents the potential for targeting metastatic cancer more effectively than conventional monotherapy.

Several clinical trials are underway to address the challenges in treating metastatic late-stage ovarian cancer. IMagyn050 (NCT03038100) evaluated the efficacy of atezolizumab, a PD-L1 inhibitor, in combination with chemotherapy and bevacizumab in stage 3/4 OC patients [83,84]. Unfortunately, it showed no statistically significant changes in progression-free survival in PD-L1-positive patients [83]. While this clinical trial has not demonstrated remarkable outcomes, it is still ongoing in phase 3, evaluating the combination of atezolizumab, an immune checkpoint inhibitor, with bevacizumab, and no severe side effects have been reported so far. LY2157299 (galunisertib) is a small molecule TGF-β inhibitor that targets type I receptor (TβRI) with decreased toxicity and no adverse effects [85]. A phase Ib trial (NCT03206177) is currently investigating a combination therapy of galunisertib with paclitaxel and carboplatin, well-known chemotherapeutic agents, against recurrent or newly diagnosed OC [86].

Bintrafusp alfa (BA) is a cutting-edge dual blockade of TGF-β and PD-L1, demonstrating the anti-tumor efficacy on metastatic OC by promoting tumor-infiltrating CD8^+^ T cells and provoking the immune response within the TME [87]. As reported in the study using the mouse models, BA treatments markedly hampered ascites development, generated inflammatory cytokines, and thus prolonged long-term survival [88]. This effect is attributed to the increased expression of CD4 and CD8 T effector memory cells and NK cells in the peritoneum [88]. In addition to BA, other dual blockades targeting signaling pathways and immunogenic factors are being explored for metastatic OC. This approach shows promise, given the multifaceted roles of metastatic signaling pathways discussed above.

### 4.3. Signaling Pathway-Targeted Therapy in Ovarian Cancer Metastasis

Given the limitations of current immunotherapy in effectively managing metastatic OC and its recurrence, there is a growing interest in targeting specific pathways that play a pivotal role in tumor progression and immunosuppressive TMEs. By integrating signaling pathway inhibitors with present immunotherapeutic approaches, researchers aim to overcome resistance mechanisms and enhance anti-cancer efficacy.

A-83-01 is an inhibitor of TGF-β, which effectively represses transcriptional alterations that occur via TGF-β1 [89]. A-83-01 treatment enables reversing EMT gene expression patterns, inhibiting MMP2 and phosphorylated SMAD2, and restraining platelet-induced invasion [90]. The in vitro study revealed that A-83-01 is more effective at reducing cell motility, invasion, and adhesion than cell proliferation, suggesting its potential as a combination therapy with immunotherapies that target cell proliferation. Additionally, A-83-01 has demonstrated anti-tumor efficacy and improved survival rates in vivo [89]. Cordycepin (3′-deoxyadenosine) is a well-established polyadenylation inhibitor with diverse capabilities, including anti-proliferative, anti-inflammatory, and anti-cancer activities as well as immune-activation properties [91]. This study elucidated that cordycepin is related to CCL5-regulated AKT/NF-κB signaling transduction in OC. AKT inactivation by downregulated CCL5 via cordycepin-induced apoptosis of OC cells and suppressed expression of nuclear NF-κB [91]. In addition, cordycepin influenced OC cell growth and autophagy in vitro [92]. CMG002, a PI3K/mTOR bifunctional inhibitor, can be synergized with paclitaxel or cisplatin and has shown prominent tumor reduction, particularly in chemoresistant OC [93]. It restrained the cancer cell proliferation and provoked apoptosis and G1 cell cycle arrest, suggesting its potential for managing chemoresistant OC [94]. Notably, CMG002 exhibits stronger anti-cancer activity at lower concentrations compared to other PI3K/mTOR inhibitors and re-sensitizes chemoresistant cells to paclitaxel or cisplatin [93,94]. Additionally, the PI3K inhibitor BKM120, when combined with the PARP inhibitor Olaparib, exhibited therapeutic effects by blocking cell proliferation, cell growth, migration, and invasion of PIK3CA mutant OC cells. This combination also regulated intraperitoneal dissemination in vivo [95]. Lastly, the mTOR kinase inhibitor CC223 caused the degradation of mTORC1 and mTORC2 complexes, effectively preventing tumor cell proliferation [96]. It also increased reactive oxygen species (ROS) production, thereby inhibiting tumor growth within the intraperitoneal cavity [94,96].

Comprehensively, signaling pathway-targeted therapies, when combined with immunotherapy, present a promising approach to addressing the challenges of metastatic OC (Table 1). By modulating metastatic pathways such as TGF-β, NF-κB, and PI3K/AKT/mTOR signaling, which are involved in tumor progression and metastasis, these combination strategies have the potential to improve clinical outcomes and provide durable responses for OC patients with fewer adverse effects.

## 5. Conclusions

OC remains a significant clinical challenge due to its high metastatic potential, complex signaling pathways, and immunosuppressive TME. Deciphering the mechanisms related to OC metastasis, particularly through TGF-β, NF-κB, and PI3K/AKT/mTOR signaling transduction, is crucial for developing effective therapeutic strategies. These pathways contribute to EMT, immune evasion, and tumor progression, facilitating the dissemination of OC cells to distant regions, especially the peritoneum. Furthermore, despite versatile immunotherapeutic approaches, the OC TME diminishes the effectiveness of conventional therapies. Within malignant ascites, cancer cells evade the immune process by the correlation between immune cells and tumors, leading to EMT and chemoresistance [98]. The compromised function of cytotoxic effector T cells weakens both innate and adaptive immunity, reinforcing the immunosuppressive characteristics of OC [99]. As a result, metastasis is exacerbated, and traditional therapies are hindered. Moreover, chemoresistance mechanisms are generally categorized into intrinsic and acquired resistance, both of which involve complex molecular and cellular adaptations [100]. Intrinsic resistance is triggered by genetic alterations and enhanced DNA repair mechanisms, reducing initial treatment efficacy [101]. In contrast, acquired resistance develops during therapy, driven by increased drug efflux, apoptosis evasion, and TME remodeling [102]. Particularly in metastatic and recurrent OC, immune evasion and adaptive resistance limit the effectiveness of treatment.

Recent advances in combination therapies targeting both signaling pathways and the immune system, such as oncolytic virus therapy, NK cell therapy, and immune checkpoint inhibitors, hold promise for overcoming these challenges. Additionally, as the aforementioned signaling pathways serve as key modulators in metastasis, combination therapies are anticipated to exhibit synergistic effects against metastatic OC. These approaches are expected to improve clinical outcomes by disrupting the metastatic cascade, reducing recurrence, and providing more durable responses with fewer side effects. Further research is required to optimize these therapies to maximize their long-term effects on patient outcomes. Also, signaling pathways are not only involved in specific tumorigenic processes but are also intricately interconnected, exerting complex effects. Thus, identifying molecules to inhibit these pathways requires delicate consideration, as targeting one pathway may inadvertently affect others, potentially causing undesirable consequences. In summary, addressing the complex networks of metastasis, immune evasion, and tumor progression offers a promising strategy to improve the prognosis and survival of OC patients. Therefore, this review highlights the mechanisms of OC metastasis and the major pathways involved, demonstrating how targeting these pathways presents a promising approach to combating OC metastasis.

## Figures and Tables

**Table 1:** Therapeutic approaches in ovarian cancer metastasis.

Type of Therapy	Related Signaling Pathway	Drug	Function	Reference
Combination immunotherapy	-	NK cell therapy with oncolytic virus	Infect cancer cells to enhance immune response and anti-tumor effect	[80]
	-	IMAgyn050	Target PD-L1 (atezolizumab) with bevacizumab	[97]
	TGF-β	LY2157299	Small molecule TGF-β inhibitor with paclitaxel and carboplatin	[85]
	TGF-β	Bintrafusp alfa	Block both TGF-β and PD-L1 to promote immune reactions, including CD4, CD8, and NK T cells	[87]
Signaling pathway-targeted therapy	TGF-β	A-83-01	Suppress EMT, MMP2, and pSMAD2 to regulate cell invasion and adhesion	[89,90]
	AKT/NF-κB	Cordycepin	Modulate cell proliferation, inflammation, anti-cancer effect as a polyadenylation inhibitor	[91]
	PI3K/mTOR	CMG002	Induce apoptosis and G1 cell cycle arrest to address chemoresistance	[94]
	PI3K	BKM120	Repress cell growth, migration, and invasion with PARP inhibitor Olaparib	[95]
	mTOR	CC223	Degrade mTORC complexes to inhibit cell proliferation and upregulate ROS generation	[94,96]

IMagyn050, NCT03038100; LY2157299, galunisertib, NCT03206177; TGF-β, transforming growth factor-β; NF-κB, nuclear factor of kappa-light chain of enhancer-activated B cells; PI3K, phosphoinositide 3-kinase; AKT, protein kinase B; mTOR, mammalian target of rapamycin; PD-L1, programmed cell death ligand; EMT, epithelial-to-mesenchymal transition; NK, natural killer; MMP, matrix metalloproteinases; pSMAD, phosphorylated suppressor of mothers against decapentaplegic; PARP, poly (ADP-ribose) polymerase; ROS, reactive oxygen species.

## Data Availability

Not applicable.

## Abbreviations

The following abbreviations are used in this manuscript:

TME	Tumor microenvironment
ECM	Extracellular matrix
TGF-β	Transforming growth factor-β
EMT	Epithelial-to-mesenchymal transition
SMAD	Suppressor of mothers against decapentaplegic
PI3K	Phosphoinositide 3-kinase
mTOR	Mammalian target of rapamycin
MAPK	Mitogen-activated protein kinase
ERK	Extracellular signal-regulated kinase
NF-κB	Nuclear factor of kappa-light chain of enhancer-activated B cells
TAMs	Tumor-associated macrophages
TNF-α	Tumor necrosis factor-alpha
PMCs	Peritoneal mesothelial cells
CAFs	Cancer-associated fibroblasts
Tregs	Regulatory T cells
MDSCs	Myeloid-derived suppressor cells
NK	Natural killer
CTLs	Cytotoxic T lymphocytes
MMPs	Matrix metalloproteinases
VEGF	Vascular endothelial growth factor
HGF	Hepatocyte growth factor
CTLA-4	Cytotoxic T lymphocyte-associated protein 4
PD-1	Programmed cell death
PD-L1	Programmed cell death ligand
VSV	Vesicular stomatitis virus
TβRI	TGF-β receptor type I
BA	Bintrafusp alfa
PARP	Poly (ADP-ribose) polymerase
ROS	Reactive oxygen species
CTC	Circulating tumor cell
MSCs	Mesenchymal stem cells
RTK	Receptor tyrosine kinase
PIP2	Phosphatidylinositol 4,5-bisphosphate
PIP3	Phosphatidylinositol (3,4,5)-triphosphate
PTEN	Phosphatase and tensin homolog
INPP4B	Inositol polyphosphate 4-phosphatase B
PDK1	Phosphoinositide-dependent protein kinase 1
HRD	Homologous recombination deficiency

## References

[1] Momenimovahed Z., Tiznobaik A., Taheri S., Salehiniya H. (2019). Ovarian cancer in the world: Epidemiology and risk factors. Int. J. Womens Health.

[2] Mei S., Chen X., Wang K., Chen Y. (2023). Tumor microenvironment in ovarian cancer peritoneal metastasis. Cancer Cell Int..

[3] Uno K., Iyoshi S., Yoshihara M., Kitami K., Mogi K., Fujimoto H., Sugiyama M., Koya Y., Yamakita Y., Nawa A. (2022). Metastatic voyage of ovarian cancer cells in ascites with the assistance of various cellular components. Int. J. Mol. Sci..

[4] Yonemura A., Semba T., Zhang J., Fan Y., Yasuda-Yoshihara N., Wang H., Uchihara T., Yasuda T., Nishimura A., Fu L. (2024). Mesothelial cells with mesenchymal features enhance peritoneal dissemination by forming a protumorigenic microenvironment. Cell Rep..

[5] Laurent-Issartel C., Landras A., Agniel R., Giffard F., Blanc-Fournier C., Cruz E.D.S., Habes C., Leroy-Dudal J., Carreiras F., Kellouche S. (2024). Ascites microenvironment conditions the peritoneal pre-metastatic niche to promote the implantation of ovarian tumor spheroids: Involvement of fibrinogen/fibrin and αV and α5β1 integrins. Exp. Cell Res..

[6] Gutic B., Bozanovic T., Mandic A., Dugalic S., Todorovic J., Dugalic M.G., Sengul D., Detanac D.A., Sengul I., Detanac D. (2023). Preliminary outcomes of five-year survival for ovarian malignancies in profiled Serbian Oncology Centre. Clinics.

[7] Garzon S., Laganà A.S., Casarin J., Raffaelli R., Cromi A., Franchi M., Barra F., Alkatout I., Ferrero S., Ghezzi F. (2020). Secondary and tertiary ovarian cancer recurrence: What is the best management?. Gland. Surg..

[8] Song M., Cui M., Liu K. (2022). Therapeutic strategies to overcome cisplatin resistance in ovarian cancer. Eur. J. Med. Chem..

[9] Elies A., Rivière S., Pouget N., Becette V., Dubot C., Donnadieu A., Rouzier R., Bonneau C. (2018). The role of neoadjuvant chemotherapy in ovarian cancer. Expert. Rev. Anticancer. Ther..

[10] Chen C., Ge X., Zhao Y., Wang D., Ling L., Zheng S., Ding K., Wang J., Sun L. (2020). Molecular Alterations in Metastatic Ovarian Cancer From Gastrointestinal Cancer. Front. Oncol..

[11] Cui M., Liu Y., Cheng L., Li T., Deng Y., Liu D. (2022). Research progress on anti-ovarian cancer mechanism of miRNA regulating tumor microenvironment. Front. Immunol..

[12] Dai W., Zhou J., Chen T. (2024). Unraveling the extracellular vesicle network: Insights into ovarian cancer metastasis and chemoresistance. Mol. Cancer.

[13] Baba A.B., Rah B., Bhat G.R., Mushtaq I., Parveen S., Hassan R., Hameed Zargar M., Afroze D. (2022). Transforming growth factor-beta (TGF-β) signaling in cancer-A betrayal within. Front. Pharmacol..

[14] Jinesh G.G., Brohl A.S. (2022). Classical epithelial-mesenchymal transition (EMT) and alternative cell death process-driven blebbishield metastatic-witch (BMW) pathways to cancer metastasis. Signal Transduct. Target. Ther..

[15] Sicard A.A., Dao T., Suarez N.G., Annabi B. (2021). Diet-Derived Gallated Catechins Prevent TGF-β-Mediated Epithelial-Mesenchymal Transition, Cell Migration and Vasculogenic Mimicry in Chemosensitive ES-2 Ovarian Cancer Cells. Nutr. Cancer.

[16] Zhou J., Jiang W., Huang W., Ye M., Zhu X. (2020). Prognostic Values of Transforming Growth Factor-Beta Subtypes in Ovarian Cancer. BioMed Res. Int..

[17] Deng Z., Fan T., Xiao C., Tian H., Zheng Y., Li C., He J. (2024). TGF-β signaling in health, disease, and therapeutics. Signal Transduct. Target. Ther..

[18] Ali S., Rehman M.U., Yatoo A.M., Arafah A., Khan A., Rashid S., Majid S., Ali A., Ali M.N. (2023). TGF-β signaling pathway: Therapeutic targeting and potential for anti-cancer immunity. Eur. J. Pharmacol..

[19] Panwar V., Singh A., Bhatt M., Tonk R.K., Azizov S., Raza A.S., Sengupta S., Kumar D., Garg M. (2023). Multifaceted role of mTOR (mammalian target of rapamycin) signaling pathway in human health and disease. Signal Transduct. Target. Ther..

[20] Glaviano A., Foo A.S., Lam H.Y., Yap K.C., Jacot W., Jones R.H., Eng H., Nair M.G., Makvandi P., Geoerger B. (2023). PI3K/AKT/mTOR signaling transduction pathway and targeted therapies in cancer. Mol. Cancer.

[21] Baghban R., Roshangar L., Jahanban-Esfahlan R., Seidi K., Ebrahimi-Kalan A., Jaymand M., Kolahian S., Javaheri T., Zare P. (2020). Tumor microenvironment complexity and therapeutic implications at a glance. Cell Commun. Signal..

[22] Harrington B.S., Annunziata C.M. (2019). NF-κB signaling in ovarian cancer. Cancers.

[23] Kaltschmidt B., Witte K.E., Greiner J.F., Weissinger F., Kaltschmidt C. (2022). Targeting NF-κB signaling in cancer stem cells: A narrative review. Biomedicines.

[24] Yin N., Li X., Zhang X., Xue S., Cao Y., Niedermann G., Lu Y., Xue J. (2024). Development of pharmacological immunoregulatory anti-cancer therapeutics: Current mechanistic studies and clinical opportunities. Signal Transduct. Target. Ther..

[25] Siminiak N., Czepczyński R., Zaborowski M.P., Iżycki D. (2022). Immunotherapy in ovarian cancer. Arch. Immunol. Ther. Exp..

[26] Shi X., Wang X., Yao W., Shi D., Shao X., Lu Z., Chai Y., Song J., Tang W., Wang X. (2024). Mechanism insights and therapeutic intervention of tumor metastasis: Latest developments and perspectives. Signal Transduct. Target. Ther..

[27] Dunbar K.J., Efe G., Cunningham K., Esquea E., Navaridas R., Rustgi A.K. (2024). Regulation of metastatic organotropism. Trends Cancer.

[28] Gao Y., Bado I., Wang H., Zhang W., Rosen J.M., Zhang X.H.-F. (2019). Metastasis organotropism: Redefining the congenial soil. Dev. Cell.

[29] Ford C.E., Werner B., Hacker N.F., Warton K. (2020). The untapped potential of ascites in ovarian cancer research and treatment. Br. J. Cancer.

[30] Pal S., Bhowmick S., Sharma A., Sierra-Fonseca J.A., Mondal S., Afolabi F., Roy D. (2023). Lymphatic vasculature in ovarian cancer. Biochim. Biophys. Acta BBA Rev. Cancer.

[31] Szczerba A., Śliwa A., Pieta P.P., Jankowska A. (2022). The role of circulating Tumor cells in Ovarian Cancer Dissemination. Cancers.

[32] Yousefi M., Dehghani S., Nosrati R., Ghanei M., Salmaninejad A., Rajaie S., Hasanzadeh M., Pasdar A. (2020). Current insights into the metastasis of epithelial ovarian cancer-hopes and hurdles. Cell. Oncol..

[33] Pascual-Antón L., Cardeñes B., Sainz de la Cuesta R., González-Cortijo L., López-Cabrera M., Cabañas C., Sandoval P. (2021). Mesothelial-to-mesenchymal transition and exosomes in peritoneal metastasis of ovarian cancer. Int. J. Mol. Sci..

[34] Ramos C., Gerakopoulos V., Oehler R. (2024). Metastasis-associated fibroblasts in peritoneal surface malignancies. Br. J. Cancer.

[35] Li K., Shi H., Zhang B., Ou X., Ma Q., Chen Y., Shu P., Li D., Wang Y. (2021). Myeloid-derived suppressor cells as immunosuppressive regulators and therapeutic targets in cancer. Signal Transduct. Target. Ther..

[36] Rakina M., Kazakova A., Villert A., Kolomiets L., Larionova I. (2022). Spheroid formation and peritoneal metastasis in ovarian cancer: The role of stromal and immune components. Int. J. Mol. Sci..

[37] Xu T., Yu S., Zhang J., Wu S. (2021). Dysregulated tumor-associated macrophages in carcinogenesis, progression and targeted therapy of gynecological and breast cancers. J. Hematol. Oncol..

[38] Li C., Jiang P., Wei S., Xu X., Wang J. (2020). Regulatory T cells in tumor microenvironment: New mechanisms, potential therapeutic strategies and future prospects. Mol. Cancer.

[39] Wang X., Xue X., Pang M., Yu L., Qian J., Li X., Tian M., Lyu A., Lu C., Liu Y. (2024). Epithelial–mesenchymal plasticity in cancer: Signaling pathways and therapeutic targets. MedComm.

[40] Huang Y., Hong W., Wei X. (2022). The molecular mechanisms and therapeutic strategies of EMT in tumor progression and metastasis. J. Hematol. Oncol..

[41] Chou M.-Y., Yang M.-H. (2021). Interplay of immunometabolism and epithelial–mesenchymal transition in the tumor microenvironment. Int. J. Mol. Sci..

[42] Wang X., Eichhorn P.J.A., Thiery J.P. (2023). TGF-β, EMT, and Resistance to Anti-Cancer Treatment. Seminars in Cancer Biology.

[43] Sun L., Xing J., Zhou X., Song X., Gao S. (2024). Wnt/β-catenin signalling, epithelial-mesenchymal transition and crosslink signalling in colorectal cancer cells. Biomed. Pharmacother..

[44] Yang Y., Ye W.-L., Zhang R.-N., He X.-S., Wang J.-R., Liu Y.-X., Wang Y., Yang X.-M., Zhang Y.-J., Gan W.-J. (2021). The role of TGF-β signaling pathways in cancer and its potential as a therapeutic target. Evid.-Based Complement. Altern. Med..

[45] Yuan Z., Li Y., Zhang S., Wang X., Dou H., Yu X., Zhang Z., Yang S., Xiao M. (2023). Extracellular matrix remodeling in tumor progression and immune escape: From mechanisms to treatments. Mol. Cancer.

[46] Peltanova B., Raudenska M., Masarik M. (2019). Effect of tumor microenvironment on pathogenesis of the head and neck squamous cell carcinoma: A systematic review. Mol. Cancer.

[47] Hao Y., Baker D., Ten Dijke P. (2019). TGF-β-mediated epithelial-mesenchymal transition and cancer metastasis. Int. J. Mol. Sci..

[48] Xue V.W., Chung J.Y.-F., Córdoba C.A.G., Cheung A.H.-K., Kang W., Lam E.W.-F., Leung K.-T., To K.-F., Lan H.-Y., Tang P.M.-K. (2020). Transforming growth factor-β: A multifunctional regulator of cancer immunity. Cancers.

[49] Zhang T., Ma C., Zhang Z., Zhang H., Hu H. (2021). NF-κB signaling in inflammation and cancer. MedComm.

[50] Bhat G.R., Sethi I., Sadida H.Q., Rah B., Mir R., Algehainy N., Albalawi I.A., Masoodi T., Subbaraj G.K., Jamal F. (2024). Cancer cell plasticity: From cellular, molecular, and genetic mechanisms to tumor heterogeneity and drug resistance. Cancer Metastasis Rev..

[51] Oh A., Pardo M., Rodriguez A., Yu C., Nguyen L., Liang O., Chorzalska A., Dubielecka P.M. (2023). NF-κB signaling in neoplastic transition from epithelial to mesenchymal phenotype. Cell Commun. Signal..

[52] Quintero-Fabián S., Arreola R., Becerril-Villanueva E., Torres-Romero J.C., Arana-Argáez V., Lara-Riegos J., Ramírez-Camacho M.A., Alvarez-Sánchez M.E. (2019). Role of matrix metalloproteinases in angiogenesis and cancer. Front. Oncol..

[53] Chen S., Liu Y., Zhong Z., Wei C., Liu Y., Zhu X. (2023). Peritoneal immune microenvironment of endometriosis: Role and therapeutic perspectives. Front. Immunol..

[54] Mao X., Xu J., Wang W., Liang C., Hua J., Liu J., Zhang B., Meng Q., Yu X., Shi S. (2021). Crosstalk between cancer-associated fibroblasts and immune cells in the tumor microenvironment: New findings and future perspectives. Mol. Cancer.

[55] Han J., Dong L., Wu M., Ma F. (2023). Dynamic polarization of tumor-associated macrophages and their interaction with intratumoral T cells in an inflamed tumor microenvironment: From mechanistic insights to therapeutic opportunities. Front. Immunol..

[56] Rinne N., Christie E.L., Ardasheva A., Kwok C.H., Demchenko N., Low C., Tralau-Stewart C., Fotopoulou C., Cunnea P. (2021). Targeting the PI3K/AKT/mTOR pathway in epithelial ovarian cancer, therapeutic treatment options for platinum-resistant ovarian cancer. Cancer Drug Resist..

[57] Ediriweera M.K., Tennekoon K.H., Samarakoon S.R. (2019). Role of the PI3K/AKT/mTOR signaling pathway in ovarian cancer: Biological and therapeutic significance. Seminars in Cancer Biology.

[58] Mafi S., Mansoori B., Taeb S., Sadeghi H., Abbasi R., Cho W.C., Rostamzadeh D. (2022). mTOR-mediated regulation of immune responses in cancer and tumor microenvironment. Front. Immunol..

[59] Ritch S.J., Telleria C.M. (2022). The transcoelomic ecosystem and epithelial ovarian cancer dissemination. Front. Endocrinol..

[60] van Baal J.O., van Noorden C.J., Nieuwland R., Van de Vijver K.K., Sturk A., van Driel W.J., Kenter G.G., Lok C.A. (2018). Development of peritoneal carcinomatosis in epithelial ovarian cancer: A review. J. Histochem. Cytochem..

[61] Maioru O.-V., Radoi V.-E., Coman M.-C., Hotinceanu I.-A., Dan A., Eftenoiu A.-E., Burtavel L.-M., Bohiltea L.-C., Severin E.-M. (2023). Developments in genetics: Better management of ovarian cancer patients. Int. J. Mol. Sci..

[62] Schiliro C., Firestein B.L. (2021). Mechanisms of metabolic reprogramming in cancer cells supporting enhanced growth and proliferation. Cells.

[63] Fu Y., Zou T., Shen X., Nelson P.J., Li J., Wu C., Yang J., Zheng Y., Bruns C., Zhao Y. (2021). Lipid metabolism in cancer progression and therapeutic strategies. MedComm.

[64] Tufail M., Jiang C.-H., Li N. (2024). Altered metabolism in cancer: Insights into energy pathways and therapeutic targets. Mol. Cancer.

[65] Zhang Z., Zhanghuang C., Mi T., Jin L., Liu J., Li M., Wu X., Wang J., Li M., Wang Z. (2023). The PI3K-AKT-mTOR signaling pathway mediates the cytoskeletal remodeling and epithelial-mesenchymal transition in bladder outlet obstruction. Heliyon.

[66] Yang C., Xia B.-R., Zhang Z.-C., Zhang Y.-J., Lou G., Jin W.-L. (2020). Immunotherapy for ovarian cancer: Adjuvant, combination, and neoadjuvant. Front. Immunol..

[67] Ghisoni E., Imbimbo M., Zimmermann S., Valabrega G. (2019). Ovarian cancer immunotherapy: Turning up the heat. Int. J. Mol. Sci..

[68] Kokabu T., Tarumi Y., Aoki K., Okamura A., Aoyama K., Kataoka H., Yoriki K., Mori T. (2024). Effects of PARP Inhibitors on Subsequent Platinum-Based Chemotherapy in Patients with Recurrent Ovarian Cancer. Cancers.

[69] Anand U., Dey A., Chandel A.K.S., Sanyal R., Mishra A., Pandey D.K., De Falco V., Upadhyay A., Kandimalla R., Chaudhary A. (2023). Cancer chemotherapy and beyond: Current status, drug candidates, associated risks and progress in targeted therapeutics. Genes. Dis..

[70] Manchado E., Guillamot M., Malumbres M. (2012). Killing cells by targeting mitosis. Cell Death Differ..

[71] Qi C., Wang X., Shen Z., Chen S., Yu H., Williams N., Wang G. (2018). Anti-mitotic chemotherapeutics promote apoptosis through TL1A-activated death receptor 3 in cancer cells. Cell Res..

[72] Wang L., Wang X., Zhu X., Zhong L., Jiang Q., Wang Y., Tang Q., Li Q., Zhang C., Wang H. (2024). Drug resistance in ovarian cancer: From mechanism to clinical trial. Mol. Cancer.

[73] Alalawy A.I. (2024). Key genes and molecular mechanisms related to Paclitaxel Resistance. Cancer Cell Int..

[74] Sahoo D., Deb P., Basu T., Bardhan S., Patra S., Sukul P.K. (2024). Advancements in platinum-based anticancer drug development: A comprehensive review of strategies, discoveries, and future perspectives. Bioorg. Med. Chem..

[75] Damia G., Broggini M. (2019). Platinum resistance in ovarian cancer: Role of DNA repair. Cancers.

[76] Gauduchon T., Kfoury M., Lorusso D., Floquet A., Ventriglia J., Salaun H., Moubarak M., Rivoirard R., Polastro L., Favier L. (2023). PARP inhibitors (PARPi) prolongation after local therapy for oligo-metastatic progression in relapsed ovarian cancer patients. Gynecol. Oncol..

[77] Boussios S., Karihtala P., Moschetta M., Karathanasi A., Sadauskaite A., Rassy E., Pavlidis N. (2019). Combined strategies with poly (ADP-Ribose) polymerase (PARP) inhibitors for the treatment of ovarian cancer: A literature review. Diagnostics.

[78] Sun G., Liu Y. (2024). Efficacy and safety of PARP inhibitor maintenance therapy for ovarian cancer: A meta-analysis and trial sequential analysis of randomized controlled trials. Front. Pharmacol..

[79] Lampert E.J., Zimmer A., Padget M., Cimino-Mathews A., Nair J.R., Liu Y., Swisher E.M., Hodge J.W., Nixon A.B., Nichols E. (2020). Combination of PARP inhibitor olaparib, and PD-L1 inhibitor durvalumab, in recurrent ovarian cancer: A proof-of-concept phase II study. Clin. Cancer Res..

[80] Gebremeskel S., Nelson A., Walker B., Oliphant T., Lobert L., Mahoney D., Johnston B. (2021). Natural killer T cell immunotherapy combined with oncolytic vesicular stomatitis virus or reovirus treatments differentially increases survival in mouse models of ovarian and breast cancer metastasis. J. Immunother. Cancer.

[81] Hoogstad-van Evert J.S., Bekkers R., Ottevanger N., Jansen J.H., Massuger L., Dolstra H. (2020). Harnessing natural killer cells for the treatment of ovarian cancer. Gynecol. Oncol..

[82] Apolonio J.S., de Souza Gonçalves V.L., Santos M.L.C., Luz M.S., Souza J.V.S., Pinheiro S.L.R., de Souza W.R., Loureiro M.S., de Melo F.F. (2021). Oncolytic virus therapy in cancer: A current review. World J. Virol..

[83] Pignata S., Bookman M., Sehouli J., Miller A., Penson R.T., Taskiran C., Anderson C., Hietanen S., Myers T., Madry R. (2023). Overall survival and patient-reported outcome results from the placebo-controlled randomized phase III IMagyn050/GOG 3015/ENGOT-OV39 trial of atezolizumab for newly diagnosed stage III/IV ovarian cancer. Gynecol. Oncol..

[84] Moore K., Bookman M., Sehouli J., Miller A., Anderson C., Scambia G., Myers T., Taskiran C., Robison K., Maenpaa J. (2020). LBA31 Primary results from IMagyn050/GOG 3015/ENGOT-OV39, a double-blind placebo (pbo)-controlled randomised phase III trial of bevacizumab (bev)-containing therapy+/-atezolizumab (atezo) for newly diagnosed stage III/IV ovarian cancer (OC). Ann. Oncol..

[85] Huang C.-Y., Chung C.-L., Hu T.-H., Chen J.-J., Liu P.-F., Chen C.-L. (2021). Recent progress in TGF-β inhibitors for cancer therapy. Biomed. Pharmacother..

[86] Makker V., Green A.K., Wenham R.M., Mutch D., Davidson B., Miller D.S. (2017). New therapies for advanced, recurrent, and metastatic endometrial cancers. Gynecol. Oncol. Res. Pract..

[87] Kment J.L., Newsted D., Young S., Vermeulen M., Craig A.W. (2023). 544 Coordinated blockade of TGF-β and PD-L1 by bintrafusp alfa promotes survival in preclinical ovarian cancer models by promoting T effector memory responses. J. Immunother. Cancer.

[88] Kment J., Newsted D., Young S., Vermeulen M.C., Laight B.J., Greer P.A., Lan Y., Craig A.W. (2024). Blockade of TGF-β and PD-L1 by bintrafusp alfa promotes survival in preclinical ovarian cancer models by promoting T effector and NK cell responses. Br. J. Cancer.

[89] Yamamura S., Matsumura N., Mandai M., Huang Z., Oura T., Baba T., Hamanishi J., Yamaguchi K., Kang H.S., Okamoto T. (2012). The activated transforming growth factor-beta signaling pathway in peritoneal metastases is a potential therapeutic target in ovarian cancer. Int. J. Cancer.

[90] Guo Y., Cui W., Pei Y., Xu D. (2019). Platelets promote invasion and induce epithelial to mesenchymal transition in ovarian cancer cells by TGF-β signaling pathway. Gynecol. Oncol..

[91] Cui Z.Y., Park S.J., Jo E., Hwang I.-H., Lee K.-B., Kim S.-W., Kim D.J., Joo J.C., Hong S.H., Lee M.-G. (2018). Cordycepin induces apoptosis of human ovarian cancer cells by inhibiting CCL5-mediated Akt/NF-κB signaling pathway. Cell Death Discov..

[92] Jang H.-J., Yang K.E., Hwang I.-H., Huh Y.H., Kim D.J., Yoo H.-S., Park S.J., Jang I.-S. (2019). Cordycepin inhibits human ovarian cancer by inducing autophagy and apoptosis through Dickkopf-related protein 1/β-catenin signaling. Am. J. Transl. Res..

[93] Choi H.J., Heo J.H., Park J.Y., Jeong J.Y., Cho H.J., Park K.S., Kim S.H., Moon Y.W., Kim J.S., An H.J. (2019). A novel PI3K/mTOR dual inhibitor, CMG002, overcomes the chemoresistance in ovarian cancer. Gynecol. Oncol..

[94] Ghoneum A., Said N. (2019). PI3K-AKT-mTOR and NFκB pathways in ovarian cancer: Implications for targeted therapeutics. Cancers.

[95] Wang D., Wang M., Jiang N., Zhang Y., Bian X., Wang X., Roberts T.M., Zhao J.J., Liu P., Cheng H. (2016). Effective use of PI3K inhibitor BKM120 and PARP inhibitor Olaparib to treat PIK3CA mutant ovarian cancer. Oncotarget.

[96] Jin Z., Niu H., Wang X., Zhang L., Wang Q., Yang A. (2017). Preclinical study of CC223 as a potential anti-ovarian cancer agent. Oncotarget.

[97] Moore K.N., Pignata S. (2019). Trials in progress: IMagyn050/GOG 3015/ENGOT-OV39. A Phase III, multicenter, randomized study of atezolizumab versus placebo administered in combination with paclitaxel, carboplatin, and bevacizumab to patients with newly-diagnosed stage III or stage IV ovarian, fallopian tube, or primary peritoneal cancer. Int. J. Gynecol. Cancer.

[98] Kumar S., Acharya S., Karthikeyan M., Biswas P., Kumari S. (2024). Limitations and potential of immunotherapy in ovarian cancer. Front. Immunol..

[99] Johnson R.L., Cummings M., Thangavelu A., Theophilou G., de Jong D., Orsi N.M. (2021). Barriers to immunotherapy in ovarian cancer: Metabolic, genomic, and immune perturbations in the tumour microenvironment. Cancers.

[100] Norouzi-Barough L., Sarookhani M.R., Sharifi M., Moghbelinejad S., Jangjoo S., Salehi R. (2018). Molecular mechanisms of drug resistance in ovarian cancer. J. Cell. Physiol..

[101] Emran T.B., Shahriar A., Mahmud A.R., Rahman T., Abir M.H., Siddiquee M.F.-R., Ahmed H., Rahman N., Nainu F., Wahyudin E. (2022). Multidrug resistance in cancer: Understanding molecular mechanisms, immunoprevention and therapeutic approaches. Front. Oncol..

[102] Wu P., Gao W., Su M., Nice E.C., Zhang W., Lin J., Xie N. (2021). Adaptive mechanisms of tumor therapy resistance driven by tumor microenvironment. Front. Cell Dev. Biol..

